# Trends in EEG signal feature extraction applications

**DOI:** 10.3389/frai.2022.1072801

**Published:** 2023-01-25

**Authors:** Anupreet Kaur Singh, Sridhar Krishnan

**Affiliations:** Department of Electrical, Computer, and Biomedical Engineering, Toronto Metropolitan University, Toronto, ON, Canada

**Keywords:** machine learning, signal analysis, assistive technology, EEG, feature extraction, brain-computer interaction

## Abstract

This paper will focus on electroencephalogram (EEG) signal analysis with an emphasis on common feature extraction techniques mentioned in the research literature, as well as a variety of applications that this can be applied to. In this review, we cover single and multi-dimensional EEG signal processing and feature extraction techniques in the time domain, frequency domain, decomposition domain, time-frequency domain, and spatial domain. We also provide pseudocode for the methods discussed so that they can be replicated by practitioners and researchers in their specific areas of biomedical work. Furthermore, we discuss artificial intelligence applications such as assistive technology, neurological disease classification, brain-computer interface systems, as well as their machine learning integration counterparts, to complete the overall pipeline design for EEG signal analysis. Finally, we discuss future work that can be innovated in the feature extraction domain for EEG signal analysis.

## 1. Introduction

Electroencephalogram (EEG) signals play an important role in understanding the electrical activity associated with brain functioning and brain-related disorders. A typical EEG signal analysis pipeline is as follows: (1) data acquisition, (2) data pre-processing, (3) feature extraction, (4) feature selection, (5) model training and classification, and (6) performance evaluation. Signal analysis, when applied to the EEG, is of particular interest as the entire body's condition, as well as brain status can often be recognized when digital signal processing (DSP) and machine learning (ML) methods are applied (Sanei and Chambers, 2021).

Carlo Matteucci and Emil Du Bois-Reymond were the first individuals to establish neurophysiology, and were the first to record and display brain activity. Later, Hans Berger discovered alpha wave activity in the brain, and he was the first to use scalp electrodes to record brain activity in the form of electrical signals in the 1870s. Berger was ultimately credited with inventing and measuring the EEG signal. Kornmüller, through his research, focused on multichannel recordings, their importance, and did so by widening the brain region covered by using a higher degree of electrodes. Since its discovery, EEG analysis has brought about significant advancements in studies of diagnosis and treatment of various neurological brain conditions and the overall health of the central nervous system (CNS). It can also be used to drive home-based technologies (telehealth), prosthetics and even in the world of virtual reality and gaming (Sanei and Chambers, 2021).

**Figure 1:**
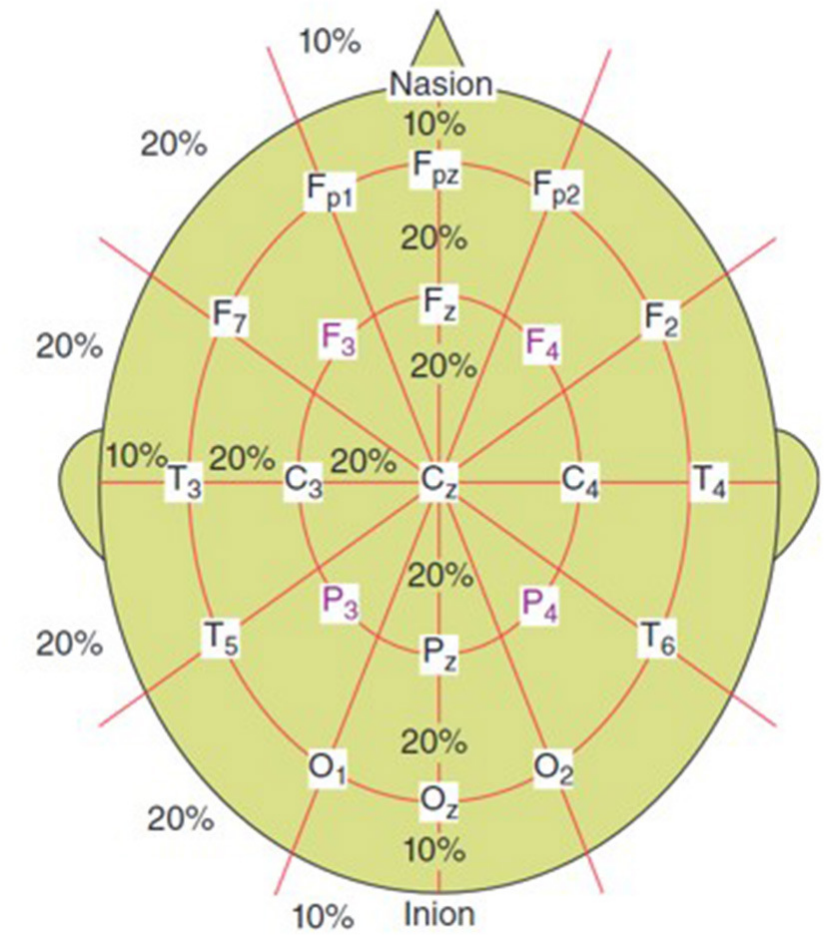
10–20 electrode setup for EEG (Sanei and Chambers, 2021).

EEG systems used for signal acquisition consist of electrodes, differential amplifiers, filters and pen-type registers. A 10–20 EEG electrode placement method is commonly used (refer to Figure 1). EEG signals are also sampled, quantized and encoded to convert them to digital form. Since the effective bandwidth of EEG signals is ~100 Hz, a minimum frequency of 200 Hz (to satisfy Nyquist criterion) is typically enough to sample the EEG for most applications (Sanei and Chambers, 2021).

### 1.1. Challenges in EEG analysis and applications

There are many applications that EEG signal analysis allows for; anywhere from disease diagnosis to even brain-computer interfaces (BCIs). A popular disorder studied heavily through EEG signal analysis is epilepsy.

Epilepsy is characterized by frequent seizures and is classified as a chronic neurological disorder. The EEG is used to identify the onset of seizures as well as for the diagnosis of epilepsy, however, this process is long and manual. Due to the manual nature, it is also subjective and thus can lead to very different diagnoses from various epileptologists. This has led to innovations in the technological realm to develop automated methods of seizure detection (Bourien et al., 2021).

EEG signal analysis is also being applied to the BCI domain, which is a rapidly growing field of research; it is an interesting field because it allows for a communication bridge between the external world and the human brain. It has been applied to assistive devices which have been used to restore movement to patients, as well as retraining patients to regain motor functionality. BCI systems function by analyzing the incoming brain waves from the EEG and converting the signal into appropriate action. There are, however, many challenges in this domain in terms of usability, training, information transfer rate, as well as technical challenges (Abdulkader et al., 2015).

Other applications of EEG include, but are not limited to, motor imagery classification, emotional classification, drug effects diagnosis, mental task diagnosis, and sleep state classification. Since large numbers of EEG channels are collected during data acquisition for these applications, there is a need for channel redundancy. There are algorithms that have been developed to assist with the channel selection of EEG signals. Channel selection assists with the reduction of computational complexity, reduce overfitting from redundant channels to improve performance, and reduce setup time in some applications. Some channel selection techniques are as follows: (1) filtering methods in which evaluation criteria are used to “filter” channels, (2) wrapping methods in which a classification algorithm is used, (3) embedded methods that select channels based on criteria generated during the learning process of classifiers, and (4) hybrid methods which combine filtering and wrapper techniques (Alotaiby et al., 2015).

### 1.2. Evolution of EEG feature extraction methods

Feature extraction is the natural next step after signal preprocessing, and is a vital step of biomedical signal analysis. It has become increasingly common to be working with big data, especially in the medical domain due to multi-hour acquisition as well as multiple channels, as is the case in EEG signal acquisition. Due to this, one of the basic goals of feature extraction is dimensionality reduction and data compaction. Essentially, this would allow one to represent their data with a smaller subset of features. This facilitates the efficient use of machine learning (ML) and artificial intelligence (AI) algorithms for classification and diagnosis applications (Subasi, 2019). Note that not all features are useful for given applications; “useful” features should, in theory, have the ability to represent the underlying signal accurately (Krishnan and Athavale, 2018; Krishnan, 2021).

Furthermore, it is important to note that EEG signals carry properties that complicate the feature extraction and signal analysis process. EEG signals are: (a) non-stationary, (b) non-linear, (c) non-Gaussian, and (d) non-short form (Alotaiby et al., 2015; Krishnan, 2021). These properties need to be accounted for in the feature extraction process for a robust end-to-end pipeline.

Feature selection is performed after feature extraction. As previously mentioned, note all features are useful for given applications, thus through the selection process, said features can be removed. Moreover, different combinations of features yield different results for pipelines; they can either affect the performance of the following ML models negatively or positively. For example, if inappropriate/inefficient features are chosen to train the model, which overall does not represent the underlying signals very well, the performance of the model would degrade. A good rule of thumb is to choose application-dependent features to represent a signal vs. generic features; this would ensure that the features would capture the patterns and behaviors of interest (Krishnan and Athavale, 2018; Subasi, 2019).

Overall, feature extraction and feature selection saves on hardware and software resources, computational time, and reduces complexity, all of which can be used to apply to the world of ML and AI-based connected healthcare and telehealth (Krishnan, 2021).

**Figure 2:**
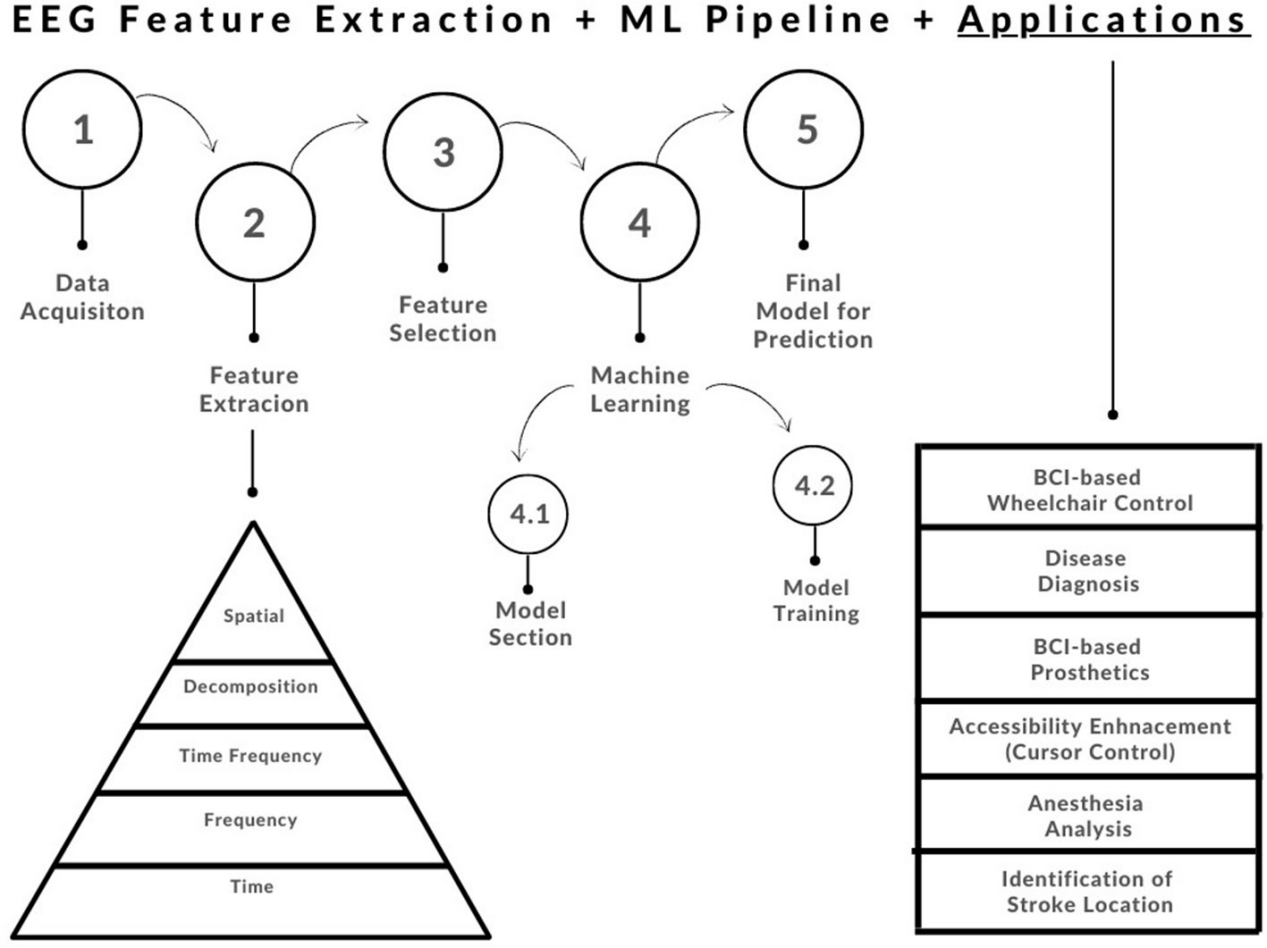
Basic feature extraction and machine learning pipeline showing the evolution of biomedical signal feature extraction techniques (Subasi, 2019).

In this paper, we will review common feature extraction methodologies that have been applied to EEG signals over the years (refer to Figure 2). This will be organized by one-dimensional feature extraction methods, vs. multi-dimensional feature extraction methods. At a high level, we will go through the following (refer to Figure 2):

- One-dimensional feature extraction techniques
  - Time domain.
  - Frequency/spectral domain.
  - Decomposition domain.
- Multi-dimensional feature extraction techniques
  - Joint time-frequency domain.
  - Spatial domain.

Note that the techniques reviewed in this paper are by no means an exhaustive list; this review serves as a starting point for analysis of EEG signals, as well as potential applications. The review work has been organized as follows: In Section 2, the authors will discuss the significance of features for machine learning. Section 3 will delve further into applications of EEG feature analysis. Section 4 will discuss common one-dimensional feature extraction techniques from the time, frequency, and decomposition domains. Section 5 will discuss multi-dimensional feature extraction methods from the joint time-frequency domain and the spatial domain. Finally, in Section 6, we will conclude the review with critical discussions, as well as potential recommendations toward future work.

## 2. Significance of features for machine learning

Following feature extraction and selection, the features are inputted and applied to ML models. These ML models are customized for specific applications, such as for classification (disease diagnosis). ML is a subset of the overall AI domain and can help with the optimization of features selected. This is done by the developer as they identify which features have a positive/negative effect on the model, and use that information to optimize the overall pipeline (Krishnan and Athavale, 2018).

The chosen application/problem must be taken into account when choosing an appropriate ML algorithm to implement. This is due to the fact that some models perform better than others for specific applications. One must also account for the inherent pros and cons of the available ML models for example some are more computationally extensive, which may not be feasible for real-time design. There are some general criteria to consider when selecting an ML algorithm: (1) Type of bio-signal, (2) Size of Feature Matrix, and (3) Availability of labeled data, just to name a few (Krishnan and Athavale, 2018). Refer to Figure 2 for a simple end-to-end feature extraction ML pipeline.

The developer can choose to either have a supervised or unsupervised ML model. Typically for medical applications, supervised models are chosen. Supervised learning refers to the availability of labeled data provided by domain experts in the field; the labeled data act as ground truth for the models to learn from during the training process.

Unsupervised learning refers to the lack of expert labeled data, where instead the algorithm studies the data to find patterns to distinguish between different classes. This type of learning is typically not used for biomedical signal data though. This is because biomedical signals are better analyzed in short-duration segments; in supervised learning, this allows for the labels to be applied to the individual segments. However, in unsupervised learning, the ML-predicted label would be applied to the full-duration signal; this is not desirable especially if there are regions-of-interest (ROIs) that require local feature extraction, not global (Krishnan and Athavale, 2018).

As previously mentioned, different sets of features yield different performance results, thus making the ML selection and training a lengthy process. The reader should be aware that the number of appropriate features is also a key point of consideration; this can lead to either model over-fitting or under-fitting issues.

## 3. Applications: Assistive technology and disease diagnosis

BCI systems can be applied to a variety of industries including medical and entertainment. In this paper, we will be focusing on potential medical applications, specifically under the umbrellas of assistive technology and disease classification. These applications can span anywhere from BCI-based prosthetics, BCI-based wheelchair control, automated disease diagnosis, assistive cursor control, and wearable devices (Rashid, 2020).

Robotic arms are one of the more common forms of BCI-based prosthetics. There are challenges that exist for disabled individuals, however, such as their loss of motion capacity that can hinder their control of these prosthetics; studies are underway to mitigate these challenges. In ideal situations, the user would be able to control in arm in all dimensions as well as carry out grasp operations (Rashid, 2020).

Brain-controlled wheelchairs (BCWs) are state-of-the-art assistive technology under neuro-rehabilitation, allowing disabled users to control a wheelchair without facing issues of fatigue, and providing them with the independence to move through various environments. BCWs allow for an improved quality of life for these users as well. This is achieved through the acquisition and analysis of the EEG signal (Fernández-Rodríguez et al., 2016).

The evaluation and diagnosis of brain diseases through the analysis of the EEG signal is another growing field. Epilepsy is the more common disease classified, but there are other neurological diseases that the EEG signal, in combination with the right features, has the potential of diagnosing. For example, brain tumors have been found to be diagnosed with the help of EEG signals, analysis of anesthesia-induced patients, as well as the locations of stoke (Song et al., 2021).

Cursor control is another popular application, allowing users that struggle to use conventional modes of cursor control an efficient alternative. Commonly, motor imagery signals are used to convey left/right/up/down operations of the cursor. Performance accuracy ranges from 70 to 95% with the use of different features (Rashid, 2020).

## 4. One-dimensional features

### 4.1. Time-domain feature extraction

Time-domain feature extraction is one of the more primitive techniques, in which the signals/data are analyzed with respect to time. This allows one to quantify how a signal is changing over time. This is especially important in an EEG signal, as they are often recorded over multi-hour timeframes. Typically, windowing and segmentation of the signal are desirable for time-domain feature extraction. This way, each window will have a local feature extracted, and the researchers will be able to view how the features change over each window. Windowing and segmentation are especially important for physiological signals as they are non-linear and non-stationary in nature (Krishnan and Athavale, 2018). In this section, various time-domain techniques specific to EEG will be explored.

#### 4.1.1. Autoregressive modeling

Autoregressive (AR) modeling uses earlier observations to create a linear regression model (Algorithm 1). When using AR modeling for feature extraction, the signal is represented by AR coefficients, which form the feature vector. This is one of the most popular forms of feature extraction in the time-dmain, and is also used in EEG-based BCI systems. This is because the technique is very conducive for data compression and low-power applications (Lawhern et al., 2012; Zhang et al., 2015; Rashid, 2020). Furthermore, AR coefficients remain invariant even in the presence of scaling changes in the data (Lawhern et al., 2012). There are however challenges with determining model order; if the order is low, it will not represent the data accurately, but if it is too high, noise increases (Rashid, 2020).

**Algorithm 1:** AR modeling feature extraction (Lawhern et al., 2012; Zhang et al., 2015; Chai, 2017).

1. **Result**: AR model coefficients 2. Import and preprocess the EEG signal 3. Segment the signal using method of choice 4. Use Equation (1) to recursively solve for the AR coefficients 5. Form the feature vector

One case study focused on the analysis of EEG signals through AR modeling to evaluate driver fatigue. EEG is widely considered as a reliable method of fatigue detection. The dataset used in this study consisted of data from 43 healthy participants from ages 18–55. Baseline EEG and subjective levels of fatigue assessment were taken, which were followed by a simulated driving task, after which another EEG measure and post-subjective levels of fatigue were measured (Chai, 2017).

(1) $$\begin{array}{l} \hat{s\left(t\right)}=\;\overset{P}{\underset{k=1}{\sum }}a\left(k\right)ŝ\left(t-k\right)+e\left(t\right), \end{array}$$

where $\hat{s\left(t\right)}$ represents the segmented EEG data, *P* is the order of the model, *e(t)* is the white noise, and *a(k)* represents the AR coefficients to be estimated (Chai, 2017).

#### 4.1.2. Fractal dimension via Higuichi algorithm

Another interesting application of EEG signal analysis is for emotion classification in BCI systems (Algorithm 2). Specifically, in this study, calm, angry and happy emotional states were studied. The data was collected from 10 subjects in real time. Video clips of 2 min each were taken while the subjects were undergoing different emotions. Fractal dimension (FD) is an index that measures signal complexity through mathematical means. The Higuichi FD algorithm is outlined in the following equation (Kaur et al., 2018):

(2) $$\begin{array}{l} FD_{j}t=\frac{\left(\sum_{i-1}^{A-jt}|X\left(j+it\right)-X\left(j+{\left(i-1\right)}^{*}t\right)|\right)\frac{A-1}{A-j}}{t}, \end{array}$$

where *X(1):X(N)* are the finite time-series samples, and *j*=*1:t* denotes the initial time to the interval time (Kaur et al., 2018).

**Algorithm 2:** Fractal dimension (FD) *via* Higuchi algorithm (Kaur et al., 2018).

1. **Result**: *FD*_*j*_*t* feature vector 2. Import and preprocess the EEG signal 3. Segment the data into 1-s intervals 4. Apply Equation (2) to apply the Higuchi FD algorithm to the EEG signal 5. Form the feature vector from *FD*_*j*_*t*

#### 4.1.3. Statistical features

Statistical feature extraction is by far one of the lesser complex methods in the time-domain (Algorithm 3). With the growing popularity of statistical programming languages, this becomes even easier with the use of native, built-in functions.

**Algorithm 3:** Statistical feature extraction (Picard et al., 2001).

1. **Result**: Statistical feature vector 2. Import and preprocess the EEG signal 3. Segment the signals 4. Use Equations (3–8) to extract the local statistical features from each EEG segment 5. Form the feature vector

One application of statistical feature extraction with EEG signals is embedding emotional intelligence into machine intelligence human-computer interaction (HCI) systems. One such study focused on classification of emotional states (subject-specific) and did so by collecting EEG data from singular subjects over multiple weeks. Thus, this is a subject-specific classification that can be expanded upon for person-independent analysis. The proposed features in the study are as follows (Picard et al., 2001):

1. Mean (raw signal)
  (3) $$\begin{array}{l} \mu_{X}=\frac{1}{N}\overset{N}{\underset{n=1}{\sum }}X_{n}, \end{array}$$
  where *X*_*n*_ represents the value of the *n*th sample of the raw signal and *n* = *1:N* data points in the raw signal.
2. Standard deviation (STD) (raw signal)
  (4) $$\begin{array}{l} \sigma_{X}={\left(\frac{1}{N-1}\overset{N}{\underset{n=1}{\sum }}{\left(X_{n}-\;\mu_{X}\right)}^{2}\right)}^{\frac{1}{2}} \end{array}$$
3. Mean of absolute values of first differences (raw signal)
  (5) $$\begin{array}{l} \delta_{X}=\frac{1}{N-1}\overset{N-1}{\underset{n=1}{\sum }}|X_{n+1}-X_{n}| \end{array}$$
4. Mean of absolute values of first differences (normalized signal)
  (6) $$\begin{array}{l} {\tilde{\delta }}_{X}=\frac{1}{N-1}\overset{N-1}{\underset{n=1}{\sum }}|{\tilde{X}}_{n+1}-{\tilde{X}}_{n}|=\;\frac{\delta_{X}}{\sigma_{X}}, \end{array}$$
  where ${\tilde{X}}_{n}$ is the normalized signal.
5. Mean of absolute values of second differences (raw signal)
  (7) $$\begin{array}{l} \gamma_{X}=\frac{1}{N-2}\overset{N-2}{\underset{n=1}{\sum }}|X_{n+2}-X_{n}| \end{array}$$
6. Mean of absolute values of second differences (normalized signal)
  (8) $$\begin{array}{l} {\tilde{\gamma }}_{X}=\frac{1}{N-2}\overset{N-2}{\underset{n=1}{\sum }}|{\tilde{X}}_{n+2}-{\tilde{X}}_{n}| \end{array}$$

#### 4.1.4. Detrended fluctuation analysis

Disease diagnosis is a huge domain in the signal analysis realm (Algorithm 4). Popularly, epilepsy is studied with the use of EEG signals, but there are other neurological diseases where EEG signal analysis can come in handy for analysis, major depressive disorder (MDD) being one of them (Mumtaz et al., 2015).

**Algorithm 4:** Detrended fluctuation analysis (Mumtaz et al., 2015)

1. **Result**: DFA feature vector 2. Import and preprocess the EEG signal 3. Remove EEG artifacts using EEGLAB 4. Segment the signal into 1-min epochs in both EO and EC conditions 5. Apply Equation (9) to obtain the scaling exponents 6. Form the feature vector

**Figure 3:**
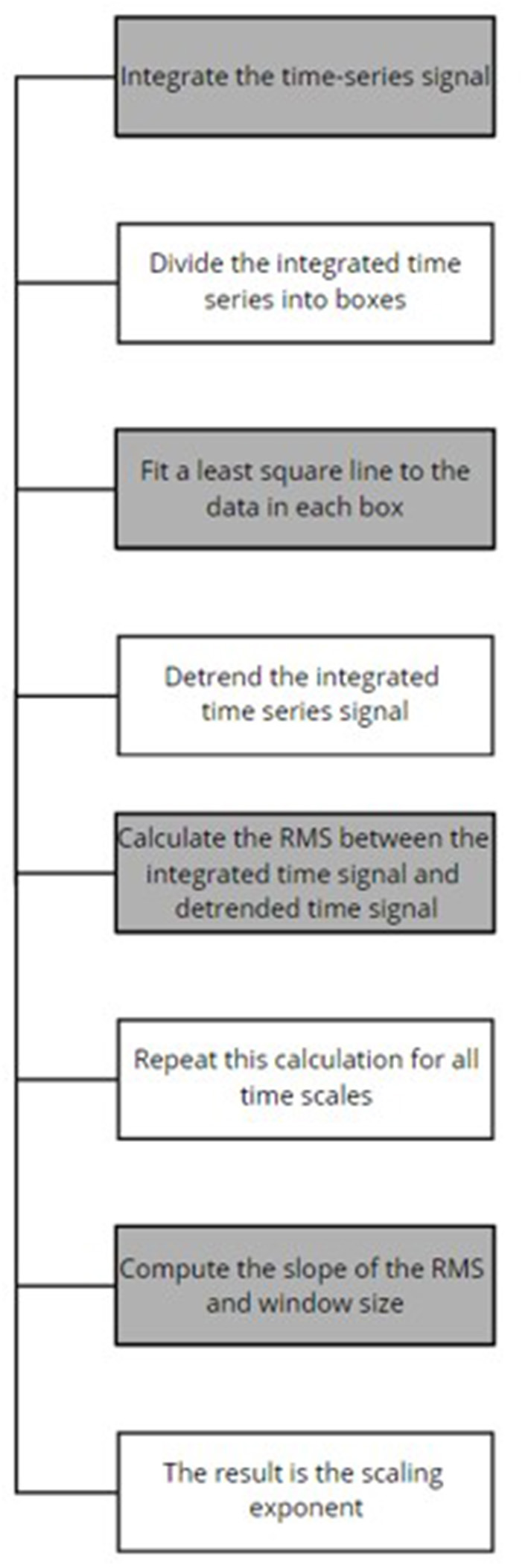
DFA process (Mumtaz et al., 2015).

The data acquired included eyes closed (EC) and eyes opened (EO) conditions from both healthy and MDD patients from the Hospital Universiti Sains Malaysia (HUMS). The data was amplified by a 24 E amplifier, sampling rate of 256 Hz was used and a bandpass filter for 0.5–70 Hz was applied. A notch filter was also used to remove the powerline interference. Detrended fluctuation analysis (DFA) was performed at the feature extraction stage to obtain the scaling exponents; refer to Figure 3 for the outlined DFA process. DFA is used to observe the presence or absence of long-range temporal correlations (LRTC) in the EEG data. DFA is computed as follows (Mumtaz et al., 2015):

(9) $$\begin{array}{l} F\left(n\right)=\sqrt{\frac{1}{N}\overset{N}{\underset{k=1}{\sum }}{\left[y\left(k\right)-y_{n}\left(k\right)\right]}^{2}}, \end{array}$$

where *N* is the length of the time-series signal, *y(k)* is the cumulative sum of the signal, and *y*_*n*_(*k*) is the resultant piecewise sequence of straight-line fits (Mumtaz et al., 2015).

### 4.2. Frequency-domain feature extraction

**Figure 4:**
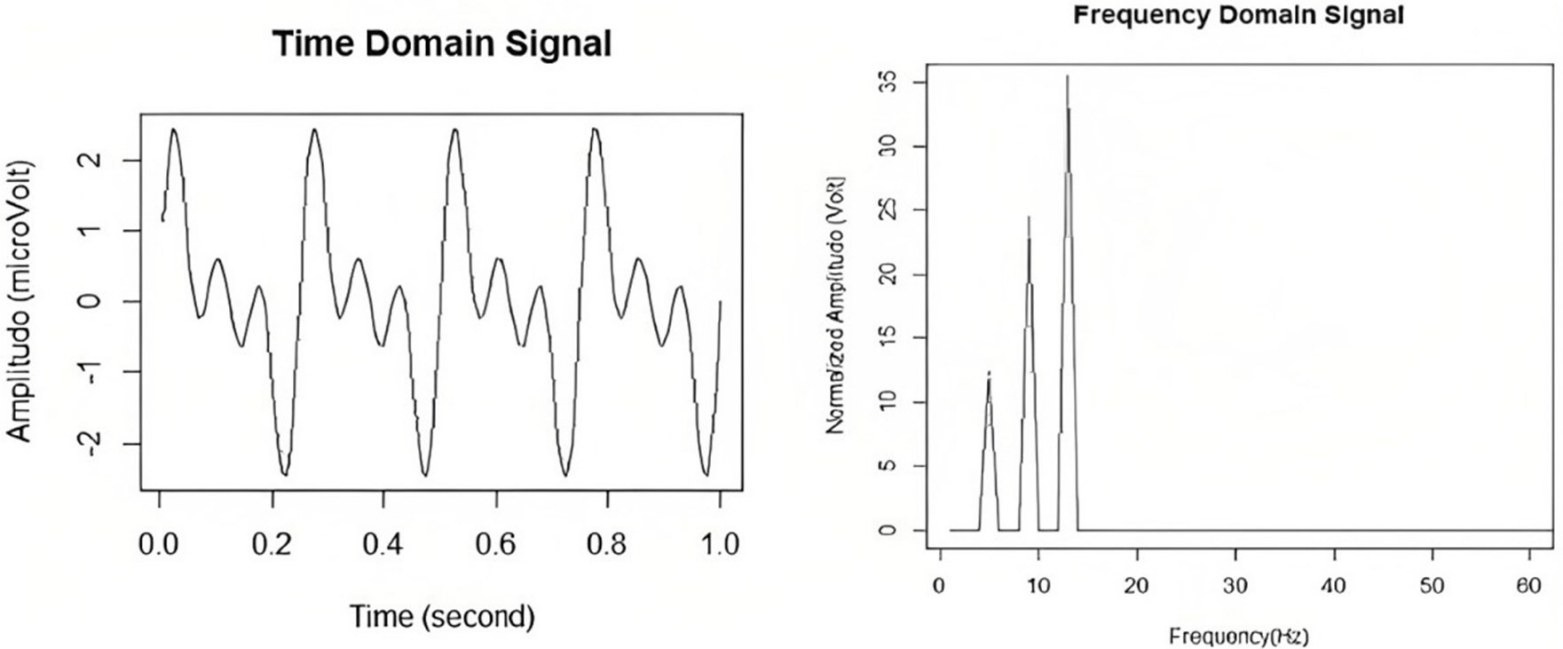
EEG signals in the time and in the frequency domain. Taken from Delimayanti et al. (2020).

The frequency domain analysis techniques focus on features that can be extracted from the sinusoids that make up the data. This is typically done by conversion from the time-domain to the frequency domain first, before further analysis can be done. Please refer to Figure 4 for a visual of a time domain signal with its frequency domain counterpart. In this section, various frequency-domain techniques specific to EEG will be explored.

#### 4.2.1. Fourier transform

It has been found that the alpha, delta, theta, and beta bands of the EEG signal operate within specific frequency bands (Algorithm 5). Thus, the analysis of the frequency spectrum of an EEG signal is important as it can help identify these bands, and classify the brainwaves. One way to do this is through fast Fourier transform (FFT) feature extraction. The data used in this study was taken from the Sleep-EDF dataset, which consists of two channels of data. 3000 FFT features were extracted (Delimayanti et al., 2020).

**Algorithm 5:** Fourier transform (Delimayanti et al., 2020).

1. **Result**: FFT feature vector 2. Import and preprocess the EEG signal 3. Segment the signal into equally-timed epochs (i.e. 30 s epochs) 4. Generate the frequency spectra of each epoch using the FFT 5. Form the feature vector

#### 4.2.2. Power spectral density

The power spectral density (PSD) is a very powerful tool in the frequency domain; from both the PSD and the normalized PSD, a variety of other features can be extracted (Algorithm 6). Some such features are the intensity weighted mean frequency (IWMF), intensity weighted bandwidth (IWBW), the spectral edge frequency (SEF), and more (Boonyakitanont et al., 2020).

**Algorithm 6:** Power spectral density (Chakladar and Chakraborty, 2018).

**Figure 5:**
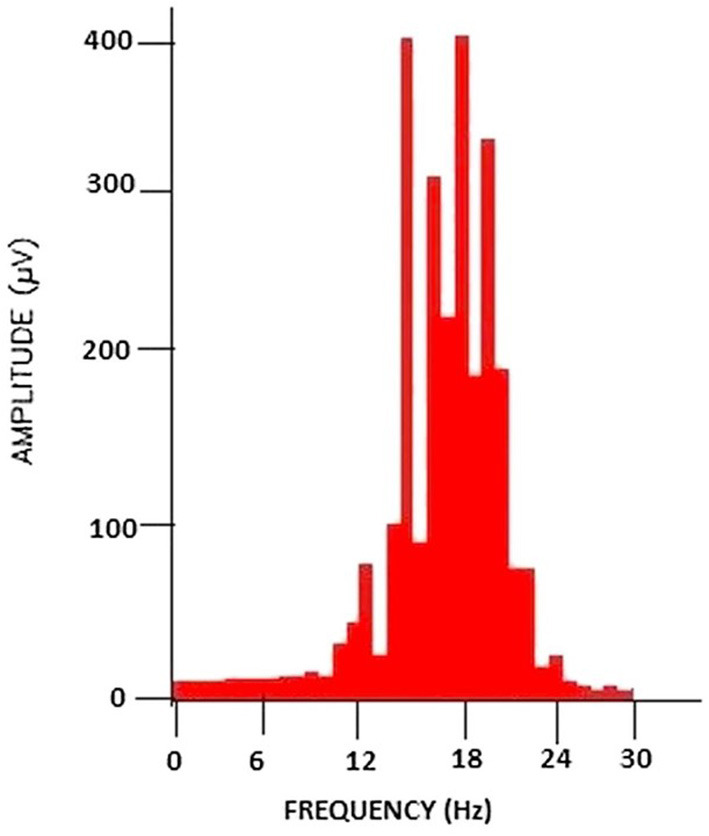
PSD analysis of the filtered brain signal. Taken from Chakladar and Chakraborty (2018).

1. **Result**: PSD feature vector 2. Import and preprocess the EEG signal 3. Filter the signal to within the frequency range of: 16 Hz (low cutoff frequency) and 24 Hz (high cutoff frequency)—refer to Figure 5 4. Extract the power spectrum features of the filtered signal 5. Form the feature vector

One case study using this method of feature extraction is focused on the application of cursor movement in BCI systems. It was shown that PSD techniques led to an increased accuracy compared to previous methods of cursor movement. Some of the data used in this study was taken from Kaggle which consisted of data from 12 subjects, in which motor imagery EEG data was collected. Thirty-two channels were used, and the signals were sampled at 500 Hz (Chakladar and Chakraborty, 2018).

#### 4.2.3. Band power

Another popular EEG-BCI application is wheelchair control (Algorithm 7). Each of these systems require a feature extraction stage to function. One research group studied the detection of stimulus frequencies through the total band power (BP) of the steady-state visual evoked potentials (SSVEPs). The band power for each stimulus frequency is estimated as follows (Mandel et al., 2009):

(10) $$\begin{array}{l} {\hat{P}}_{k,l}=\;||X_{k}^{T}s_{l}|{|}^{2}, \end{array}$$

where *X* is an SSVEP model with noise excluded, and *s*_*l*_ is the *l*th channel signal (Mandel et al., 2009).

**Algorithm 7:** Band power (Mandel et al., 2009).

1. **Result**: Band power feature vector 2. Import and preprocess the EEG signal 3. Perform segmentation for each of the channels into individual SSVEPs 4. Apply Equation (10) 5. Form the feature vector

After the power is estimated, a linear classifier is applied to classify the frequency of which the subject was focusing on. The minimum accuracy achieved with this system was 93.61% during wheelchair movement. It was also found that stressful situations for the subject did not hinder the performance significantly (Mandel et al., 2009).

#### 4.2.4. Hilbert-Huang transform

Although epilepsy is one of the more common diseases studied through the use of EEG, there are various others that are promising to further the development in the EEG-disease diagnosis realm (Algorithm 8). Schizophrenia is a brain disorder in which there is still a lack of overall understanding, which also makes diagnosis difficult. However, it has recently been shown that, for patients suffering schizophrenia, their left hemispheres of the brain show impairment. This became a starting ground for EEG diagnostic research (Azlan and Low, 2014).

**Algorithm 8:** Hilbert-Huang transform (Azlan and Low, 2014).

1. **Result**: HHT feature vector 2. Import and preprocess the EEG signal 3. Perform empirical mode decomposition to decompose the data into the intrinsic mode functions (IMFs)—sifting process 4. Apply Equation (11) to find the instantaneous frequencies from the IMFs (apply to each). 5. Perform the Hilbert spectrum 6. Form the feature vector

Some feature extraction techniques have been studied for this application, but the one this paper will review is the Hilbert-Huang transform (HHT). Essentially, the FFT of the input signal is taken. Then, the FFT coefficients that correspond to negative frequencies are zeroed, and then the inverse FFT is taken. The general definition of the Hilbert Transform (HT) is as follows (Azlan and Low, 2014):

(11) $$\begin{array}{l} y\left(t\right)=\frac{1}{\pi }PV\int_{-\infty }^{\infty }\frac{x\left(t^{\prime }\right)}{t-t^{\prime }}dt, \end{array}$$

where *PV* is the Cauchy Principal Value (Azlan and Low, 2014).

The data used in this study was obtained from the UCI ML repository for genetic predisposition to alcoholism. Five subjects were taken from the control group and the alcoholic group, for a total of 10 (Azlan and Low, 2014).

### 4.3. Decomposition-domain feature extraction

Decomposition feature extraction is useful as it allows for simultaneous filtering of the signal as well. The basic premise is as follows: (1) decompose the signal using a method of choice, (2) select the desired components, and (3) reject the undesirable components. This also leads to data compaction, making it ideal for more modern applications. In this section, various decomposition methods specific to EEG applications will be reviewed.

#### 4.3.1. Adaptive Hermite decomposition

Adaptive Hermite decomposition (AHD) uses Hermite functions to find the applications for analysis of signals, in this case EEG signals (Algorithm 9). In this study, the Hermite functions (HFs) in question are adaptively selected for the EEG signals with the use of evolutionary optimization algorithms (EOAs). Many road accidents are caused by impaired driving due to drowsiness. Thus, a quick and efficient drowsiness detection test has been of interest. This case study focuses on an ML drowsiness detection mechanism using AHD and Hermite functions (Taran and Bajaj, 2018).

**Algorithm 9:** AHD algorithm (Taran and Bajaj, 2018).

1. **Result**: HC-based statistical feature vector 2. Import and preprocess the EEG signal 3. Perform AHD using Equations (12), (13) 4. Extract the statistical features from the HCs (first quartile, range, median and energy) 5. Form the feature vector

The data was taken from the MIT/BIH polysomnographic database from 16 subjects. A sampling rate of 250 Hz. Supervised learning was employed as an expert labeled the epochs at each 30-s interval (Taran and Bajaj, 2018).

The dilation factor, *p*, and the *kth* order HF, denoted as *f*_*k,p*_(*t*), is defined as follows (Taran and Bajaj, 2018):

(12) $$\begin{array}{l} f_{k,p}\left(t\right)=\frac{1}{\sqrt{p2^{k}k!\sqrt{\pi }}}e^{-\left(\frac{t^{2}}{2p^{2}}\right)}h_{k}\left(\frac{t}{p}\right), \end{array}$$

(13) $$\begin{array}{l} c_{k}=\langle x,f_{k,p}\rangle =\;\int_{-\infty }^{\infty }x\left(t\right)f_{k,p}\left(t\right)dt,\;\left\{0\leq k\leq n-1\right\}, \end{array}$$

where $h_{k}\left(\frac{t}{p}\right)$ is the dilated form of *h*_*k*_(*t*), a Hermite polynomial, and *c*_*k*_ are the Hermite coefficients (HCs) that will be used as the basis of the features. From the HCs, statistical measures will be taken as the features; the first quartile, median, range and energy are the statistical features that will be extracted (Taran and Bajaj, 2018).

#### 4.3.2. Local characteristic-scale decomposition

Local characteristic-scale decomposition (LCSD) works to disintegrate the raw EEG data, and creates segments that convey the properties of the original signal (Rashid, 2020) (Algorithm 10). The signal is decomposed into various intrinsic scale components (ISCs), in which the instantaneous frequency of each ISC has high significance. The decomposition is performed as follows (Liu et al., 2017):

(14) $$\begin{array}{l} x\left(t\right)=\overset{n}{\underset{p=1}{\sum }}ISC_{p}\left(t\right)+u_{n}\left(t\right), \end{array}$$

where *x*(*t*) is the original signal and *u*_*n*_(*t*) is the residue (Liu et al., 2017).

**Algorithm 10:** LCSD algorithm (Liu et al., 2017).

1. **Result**: LCSD-based feature vector 2. Import and preprocess the EEG signal 3. Use Equation (14) to decompose the signal into ISCs 4. Extract frequency domain features from the ISCs 5. Form the feature vector

The data in this case study was taken from Graz University from their 2008 BCI competition dataset. The dataset includes imagination movements from the left hand, right hand, both feet and tongue. A sampling rate of 250 Hz was used and the signals were bandpass filtered between 0.5 and 100 Hz. A 50 Hz notch filter was also used to remove the powerline interference. In this case study, it's feasibility of use in a real-time BCI system was tested as well, showing its capability (Liu et al., 2017).

#### 4.3.3. The wavelet transform

The wavelet transform (WT) is a popular method of decomposition as it allows for effective use with signals that are non-stationary (Ieracitano et al., 2020). There are two versions of the WT, continuous and discrete. The following sections will review sample applications of both versions, as well their advantages and disadvantages.

##### 4.3.3.1. The continuous wavelet transform

The continuous wavelet transform (CWT) allows for a signal to be projected into the time-frequency domain (Algorithm 11). It is continuous because the translation and scale parameters of wavelets are varying continuously. The CWT can be used as an effective feature extraction technique for classification of EEG signals.

**Algorithm 11:** CWT algorithm (Ieracitano et al., 2020).

1. **Result**: CWT feature vector 2. Import and preprocess the EEG signal 3. Downsample the EEG signals from each channel to 256 Hz 4. Partition the EEG signal into non-overlapping epochs of 5 s duration 5. Compute the CWT (Equation 15) of each epoch, projecting them into the time-frequency domain. Use the Mexican hat mother wavelet function 6. Average the epochs from each channel to form a signal average epoch in time-frequency representation (TFR) 7. Further partition the epochs in the five EEG rhythms; delta band, alpha1 and alpha2 bands, theta band and beta band 8. From each EEG sub-band, extract the mean, STD, skewness, kurtosis and entropy 9. Form the feature vector

Alzheimer's disease (AD) accounts for about 60% of all dementia cases. The intermediate stage between healthy aging and AD is coined amnestic Mild Cognitive Impairment (MCI). Typically, an MCI patient that develops AD will also develop dementia in about a seven-year timeline. The detection of this disorder onset remains a challenging task that researchers are looking to automate using feature extraction and ML techniques. In this study, CWT features were used to classify AD and MCI patients from the healthy controls (healthy elderly subjects). A sampling frequency of 1024 Hz was used for the EEG signals collected, and a notch filer was applied at 50 Hz to remove the powerline interference. A bandpass filter was also applied between 0.5 and 32 Hz. The CWT is defined as follows (Ieracitano et al., 2020):

(15) $$\begin{array}{l} CWT\left(a,b\right)=\frac{1}{\sqrt{a}}\int s\left(t\right)\psi^{*}\left(\frac{t-b}{a}\right), \end{array}$$

where *a* is the dilation factor, *b* is the shifting factor, ψ is the mother wavelet function, and *CWT*(*a, b*) represents the wavelet coefficients (Ieracitano et al., 2020).

##### 4.3.3.2. The discrete wavelet transform

Another WT-based case study is focused on the development of BCI systems that would allow users to output desired characters to their computer screens through their P300 event-related potential (ERP) in their EEG signals (Algorithm 12). The L-level coefficients of the DWT, *d*^*ks*^, of the signal, *f*^*ks*^, are computed as follows (Guo et al., 2015):

(16) $$\begin{array}{l} d^{ks}=Wxf^{ks}, \end{array}$$

where *k* denotes the channel, *s* represents the trial number, and *W* is the transformation matrix. The data in this study was recorded with a sampling rate of 1,000 Hz. 800 ms long epochs were extracted starting from stimulus onset (Guo et al., 2015).

**Algorithm 12:** DWT algorithm (Guo et al., 2015).

1. **Result**: DWT feature vector 2. Import and preprocess the EEG signal 3. Determine and set the following parameters: mother wavelet, wave level, and feature number in a single channel 4. Apply Equation (16) to obtain the l-level DWT coefficients 5. Divide the data segments into the target group and non-target group 6. Compute the between-class and within-class distances for the two groups 7. Apply the Fisher criterion. Sort the output in descending order 8. Form the feature vector

#### 4.3.4. Empirical mode decomposition

The empirical mode decomposition (EMD) method of decomposing a signal allows for effective preprocessing and artifact removal (Algorithm 13). This is an important step in EEG signal analysis as EEG signals are heavily impacted by noise sources such as powerline interference and EMG noise. EMD is an ideal method to do this as it retains much of the target characteristics of the original signal (Zhang et al., 2008).

**Algorithm 13:** EMD algorithm (Zhang et al., 2008).

1. **Result**: IMFs 2. Import the EEG signal 3. Identify the local maxima and local minima in the signal 4. Connect the local maxima *via* a cubic spline curve; this becomes the upper envelope denoted as *x*_*u*_(*t*) 5. Connect the local minima by a spline curve; this becomes the lower envelope denoted as *x*_*l*_(*t*) 6. Calculate the mean value at every point of the envelope: (17) $$\begin{array}{l} m_{1}\left(t\right)=\frac{x_{u}\left(t\right)+l\left(t\right)}{2} \end{array}$$ 7. Obtain the first “prototype” IMF, *h*_1_(*t*). This is the “sifting” process: (18) $$\begin{array}{l} h_{1}\left(t\right)=x\left(t\right)-\;m_{1}\left(t\right) \end{array}$$ 8. Continue repeating the sifting process, replacing *x*(*t*) each time with the previous IMF. In this way, the IMFs act as the original data. For example: (19) $$\begin{array}{l} h_{11}\left(t\right)=h_{1}\left(t\right)-\;m_{11}\left(t\right) \end{array}$$ 9. Repeat the sifting process until you have formed an IMF, *h*_1*k*_(*t*), and denote it as *c*_1_ (20) $$\begin{array}{l} c_{1}=h_{1k}\left(t\right)=h_{1\left(k-1\right)}\left(t\right)-\;m_{1k}\left(t\right) \end{array}$$ 10. Confirm that *c*_1_ meets the IMF criteria 11. Subtract the IMF from the original signal to find the residual signal (21) $$\begin{array}{l} r_{1}\left(t\right)=x\left(t\right)-IMF \end{array}$$ 12. Repeat steps 7–11, using the previous residual function as the original signal *x(t)*. Repeat until the residual function found is a monotonic function 13. **BONUS**: This is not done in the case study, but feature extraction can be performed at this stage. For example, you can extract spectral and statistical features from the IMFs of the signal, and form a feature vector to train an ML model for applications such as classification

During the EMD procedure, the EEG signal is split into levels of intrinsic mode functions (IMFs), which are related to the frequency distribution in the signal. The IMFs are computed through a “sifting” process (Zhang et al., 2008).

An IMF is classified as an IMF if it meets the following requirements (Zhang et al., 2008):

1. count (local extrema) = count (zero crossings) ± 1.
2. average (envelope) = 0 at all time points.

## 5. Multi-dimensional feature extraction

### 5.1. Joint time-frequency domain feature extraction

Spectral and time characteristics of signals alone for feature extraction are oftentimes ineffective due to the absence of their counterparts as they neglect one another. To overcome the limitations of single domains, time-frequency analysis leverages both (Rashid, 2020). This section will review some well-known time-frequency feature extraction techniques for EEG analysis.

#### 5.1.1. Short-time Fourier transform

There is much work being done to improve the performance of motor imagery based BCIs (Algorithm 14). This is difficult as motor imagery EEGs are typically inconsistent and distorted. One study looks to enhance this with the use of the short-time Fourier transform (STFT) to convert EEG signals into 2D images, and then use the images to train and test a capsule network (Ha, 2019).

**Algorithm 14:** STFT algorithm (Ha, 2019).

1. **Result**: STFT 2D images 2. Import and preprocess the EEG signal 3. Select the desired windowing function 4. Apply Equation (22) with an overlap size of 100, and window size of 128 5. Extract the vectors within the frequency range between mu and beta waves 6. Form the 2D images

The sensorimotor rhythm (SMR) brainwave is observed after attempted or executed tasks, hence it is the brainwave most commonly used for motor imagery-based BCIs (Ha, 2019).

The STFT algorithm converts the 1D motor imagery signals from each EEG electrode into a 2D image in the time-frequency domain. The STFT is defined as follows (Haddad, 1993; Ha, 2019):

(22) $$\begin{array}{l} STFT\left(\tau ,w\right)=\int x\left(t\right)w\left(t-\tau \right)e^{-jwt}dt, \end{array}$$

where *w(t)* is the window function, and *x(t)* is the EEG signal to be transformed. Windowing functions such as the Hann and Gaussian can be used. This allows for 2D spectrogram images to be extracted (Ha, 2019).

The data used in this study was taken from the BCI competition IV 2b dataset, which was obtained from nine subjects during left-hand and right-hand motor imagery tasks. A sampling frequency of 250 Hz was used, and the signals were bandpass filtered between 0.5 and 100 Hz. A notch filter was also applied at 50 Hz. The results from this study outperformed that of standard convolutional neural network (CNN) based methods, and show feasibility for this approach to be used for classification of motor imagery EEG signals (Ha, 2019).

#### 5.1.2. S-transform

**Figure 6:**
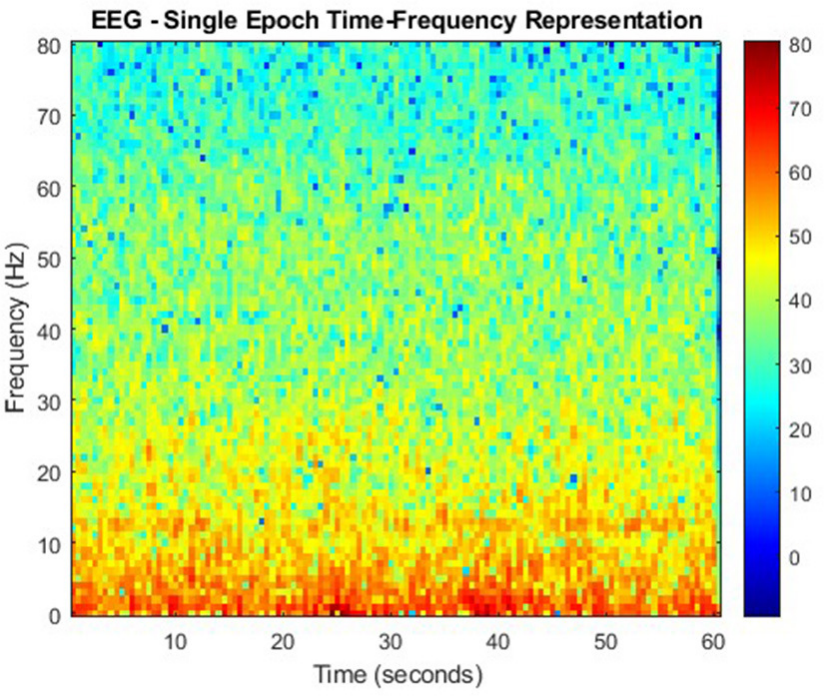
Time-frequency representation of a signal using the ST.

Continuing on the theme of EEG signal classification, it has been found that the use of the S-transform (ST) as a feature extraction technique has been effective as well (Algorithm 15). In this study, data taken from the Ward of Neurology and Strokes of the Provincial Hospital of Zielona Gora is taken. They were acquired using 16 channels, and form a complete database of neuro-disorders. This study focused on epileptic and non-epileptic subjects. A sampling frequency of 500 Hz was used and the signals were low-pass filtered with a cutoff frequency of 35 Hz. Supervised learning was employed as an expert labeled each record as epileptic or normal (Rutkowski et al., 2013). For Figure 6 the time-frequency representation of an epoch to which the S-transform has been applied.

**Algorithm 15:** ST algorithm (Rutkowski et al., 2013).

1. **Result**: ST feature vector 2. Import and preprocess the EEG signal 3. Filter the signals to the frequency range 0–100 Hz 4. Apply Equations (23), (24) 5. Use a sampling rate of 10 to achieve 11 components (each with 1,500 samples) of each channel 6. Extract the following features: mean, STD, median, mode, component energy, and component entropy, giving a total of 880 attributes 7. Form the feature vector

The S-transform can be considered a generalization of the previous STFT, but instead of a constant sized window, a scalable Gaussian window is used. The S-transform is computed as follows (Rutkowski et al., 2013):

(23) $$\begin{array}{l} S\left(t,f\right)=\int_{-\infty }^{\infty }x\left(\tau \right)g\left(\tau -t\right)e^{-j2\pi f\tau }d\tau , \end{array}$$

where *g*(τ−*t*) is the Guassian function at τ = *t* defined as (Rutkowski et al., 2013):

(24) $$\begin{array}{l} g\left(\tau -t\right)=|f|e^{-\pi {\left(\tau -t\right)}^{2}f^{2}} \end{array}$$

#### 5.1.3. Matching pursuit

Similar to the S-transform case study, this study used data taken from the Ward of Neurology and Strokes of the Provincial Hospital of Zielona Gora (Algorithm 16). The signals were acquired using 16 channels, and form a complete database of neuro-disorders. This study focused on epileptic and non-epileptic subjects. A sampling frequency of 500 Hz was used, and the signals were low-pass filtered with a cutoff frequency of 35 Hz. Supervised learning was employed as an expert labeled each record as epileptic or normal (Rutkowski et al., 2013).

**Algorithm 16:** MP algorithm (Rutkowski et al., 2013).

1. **Result**: Feature vector of weights *a*_*n*_ and atoms *g*_*n*_(*t*) 2. Import and preprocess the EEG signal 3. Find the atom with the largest inner product with the signal 4. Subtract the contribution from this atom from the signal 5. Repeat steps 3–4 until the signal is decomposed 6. Use Equation (25) to confirm that the MP algorithm is complete 7. Form the feature vector

The matching pursuit (MP) method works to identify the best matching projections of data onto a dictionary, *D*. A commonly used dictionary is based on Gabor functions. However, this method is quite greedy and computationally expensive. MP allows the signal to be represented as combination of weighted sums as follows (Rutkowski et al., 2013):

(25) $$\begin{array}{l} x\left(t\right)=\overset{\infty }{\underset{n=0}{\sum }}a_{n}g_{n}\left(t\right), \end{array}$$

where *a*_*n*_ are the weights and *g*_*n*_(*t*) are the atoms (Rutkowski et al., 2013).

### 5.2. Spatial domain feature extraction

Spatial domain feature extraction, AKA spatial filtering, is one of the most popular classification techniques for EEG signals; specifically, the common spatial pattern (CSP), a supervised spatial filter, is used. A spatial filtering method converts the brain waves into a unique space. In this unique space, the variance of one group is magnified, and a lower variance is seen in the remaining group. However, there are limitations with the pure CSP technique; due to the subject-specific optimal frequency band, it cannot achieve an ideal performance. Due to this, researchers have been developing variations of the CSP to overcome the limitations. This section will look at some of the changes that have been proposed and tested (Reddy et al., 2019; Rashid, 2020).

#### 5.2.1. Common spatio-spectral pattern

The common spatio-spectral pattern (CSSP) approach builds on the pure CSP approach by simply combining an FIR filter with the CSP algorithm (Algorithm 17). It has been shown to have improved performance vs. the purse CSP on its own (Rashid, 2020).

**Algorithm 17:** CSSP algorithm (Reddy et al., 2019).

1. **Result**: The spatial filter matrices: [*W*^(0)^ *W*^(1)^ *W*^(2)^] 2. Import and preprocess the EEG signal 3. Bandpass filter the signal to remove the mean from 1 to 20 Hz 4. Downsample the data to 250 Hz 5. Compute the thresholds of the Gaussian membership functions for each of the fuzzy classes 6. Compute $\bar{X_{i}}$: (28) $$\begin{array}{l} \bar{X_{i}}=\;\frac{\sum_{k=1}^{N_{i}}\mu_{k,i}X^{k}}{Ni}, \end{array}$$ where *N*_*i*_ is the number of trials in the ith fuzzy class. 7. Compute the covariance matrix for each fuzzy class using: (29) $$\begin{array}{l} \overset{\_}{\underset{i}{\sum }}=\;{\bar{X}}^{i}{{\bar{X}}^{i}}^{T}, \end{array}$$ where *T* denotes the transpose operation 8. Normalize the covariance matrices: (30) $$\begin{array}{l} \overset{\_}{\underset{i}{\sum }}=\;\frac{\bar{\sum }{\;}_{i}}{Tr\left(\bar{\sum }{\;}_{i}\right)}, \end{array}$$ where *Tr* denotes the trace matrix operation. 9. Compute the spatial filters matrix ***W***. Note that ***W*** must satisfy the following: (31) $$\begin{array}{l} W\overset{\_}{\underset{i}{\sum }}W^{T}=\;D_{i} \end{array}$$ (32) $$\begin{array}{l} \overset{M}{\underset{i=1}{\sum }}D_{i}=I_{NxN}\; \end{array}$$ 10 Obtain: [*W*^(0)^ *W*^(1)^ *W*^(2)^]

This particular study looked at extending the CSP algorithm to EEG state-space through fuzzy time delay, and is a novel approach. The data in this study was collected over a 5-month period, and was supposed by the National Chiao Tung University, Taiwan. This method was shown to improve the overall signal quality. In this method, a spatial filter is applied (Reddy et al., 2019):

(26) $$\begin{array}{l} \bar{x}=\underset{i}{\sum }w_{i}x_{i}, \end{array}$$

where $\bar{x}$ is the EEG signal after spatial filtering has been applied, *w*_*i*_ are the spatial filter weights, and *x*_*i*_is a row vector that contains the EEG signal from channel *i* prior to spatial filtering. The CSSP algorithm is defined as follows (Reddy et al., 2019):

(27) $$\begin{array}{l} Z^{k}=\overset{2}{\underset{\tau =0}{\sum }}\mu_{\left(\tau \right)}W^{\left(\tau \right)*}\left(\delta^{\tau }X^{k}\right)=\left[W^{\left(0\right)}\;W^{\left(1\right)}\;W^{\left(2\right)}\right]\left[\begin{array}{c} \mu_{0}X^{\left(k\right)}\\ \mu_{1}X^{\left(k-1\right)}\\ \mu_{2}X^{\left(k-2\right)} \end{array}\right], \end{array}$$

where δ^τ^ is the delay operator, μ_(τ)_ is the fuzzy membership value for τ, *W*^(τ)^ is the optimized fuzzy CSSP weights matrix, and *X*^*k*^ is the preprocessed EEG recording of the *k*th trial (Reddy et al., 2019).

After the CSSP algorithm is applied, features can be further extracted for the purposes of EEG-based Regression Problems in BCIs (Reddy et al., 2019).

#### 5.2.2. Common sparse spatio-spectral patterns

An extension of the aforementioned CSSP method, the common sparse spatio-spectral patterns (CSSSP) algorithm is a comparatively more advanced procedure in which an investigation is carried out as to where the common spectral patterns across EEG channels are located (Rashid, 2020) (Algorithm 18).

**Algorithm 18:** CSSSP algorithm (Dornhege et al., 2006).

1. **Result**: Sequence: *b*_1_*,…*,_*T*_ with *b*(1) = 1 2. Import and preprocess the EEG signal 3. Apply a casual bandpass filter from 7 to 30 Hz, containing the μ- and β- rhythms 4. Extract 500–3,500 ms after the visual stimulus was presented 5. Solve the CSSP algorithm—see Equation (33)—to obtain the sequence 6. Form the feature vector

This case study focusses on a technique that allows for optimization of spatial and spectral filters together, which enhance the discriminability rates of the multichannel EEG trials. The CSSSP algorithm will be capable of learning a global spatial-temporal FIR. Each class will have a frequency band filter and a pattern. Sparsity is introduced to restrict the complexity of the frequency filter. The CSSSP algorithm looks to find a real valued sequence *b*_1_*,…*,_*T*_ with *b*(1) = 1 (Dornhege et al., 2006):

(33) $$\begin{array}{l} s_{i,b}=s_{i}+\underset{\tau =2,\ldots ,T}{\sum }b_{\tau }s_{i}^{\tau }, \end{array}$$

where *s*_*i*_ is the signal, and $s_{i}^{\tau }$ is the signal delayed by τ time points (Dornhege et al., 2006).

#### 5.2.3. Sub-band common spatial patterns

The sub-band common spatial patterns (SBCSP) algorithm consists of first filtering the EEG signal at various sub-bands, which is followed by the calculation of traditional CSP features for each of the sub-bands (Khan et al., 2019; Rashid, 2020) (Algorithm 19).

**Algorithm 19:** SBCSP algorithm (Khan et al., 2019).

**Figure 7:**
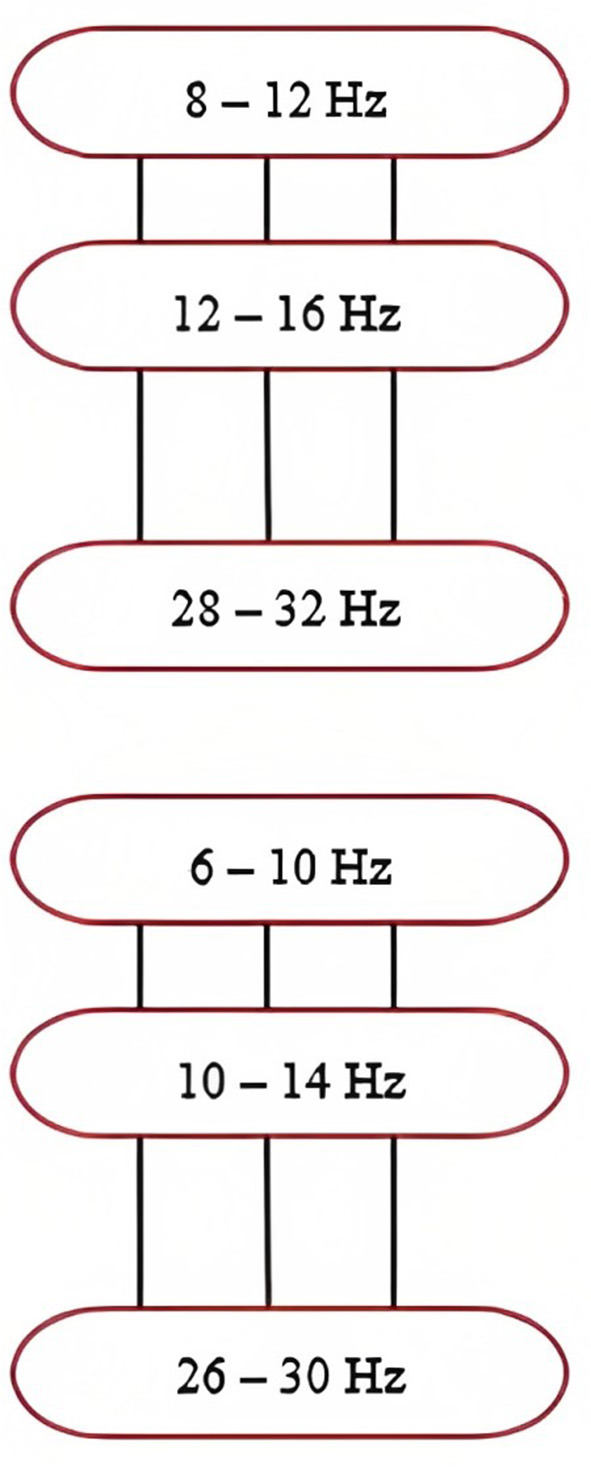
Frequency ranges of filters within the filter bank. Note that the overlapping frequencies minimize information loss. Taken from Khan et al. (2019).

1. **Result**: SBCSP feature vector 2. Import and preprocess the EEG signal 3. Apply a filter bank containing different bandpass filters for different frequency bands a. Refer to Figure 7 for the frequency bands 4. Apply the traditional CSP to each bandpass filtered signal to extract features a. Extract the maximum ration of variances b. Compute the composite spatial covariance matrix *E* (34) $$\begin{array}{l} E=\;\overset{M_{N}}{\underset{j=0}{\sum }}E_{j,n}E_{j,n}^{T}, \end{array}$$ where *M*_*N*_ represents all the trials in “*n”* classes c. Apply Equation (35) to find transformed data variance between multiclass data (35) $$\begin{array}{l} \underset{x}{\max}W\left(s\right)=\frac{s^{T}E_{c1}s}{s^{T}E_{c2}s}s.t.||s|{|}_{2}=1, \end{array}$$ where *W(s)* represents the Rayleigh quotient maximization, *s* is the spatial filter, ||*s*||_2_ is the *n*_2_ normal, and *E*_*c*1_ and *E*_*c*2_ are the covariance matrices of classes 1 and 2 5. Apply linear discriminant analysis (LDA) to each sub-band to acquire scores that show the classification capability of each band 6. Form the feature vector

The data in this study was sampled at a rate of 128 Hz. The 10–20 electrode system was used to acquire the EEG signals. The results showed that the SBCSP algorithm showed a 7% increase in accuracy when compared to other methods (Khan et al., 2019).

#### 5.2.4. Regularized CSP

Regularized CSP (RCSP) is arising as another CSP method that allows for feature extraction from selected channels (Rashid, 2020) (Algorithm 20). This method also allows for the optimization of motor imagery features, and improves classification accuracy (Jin et al., 2019).

**Algorithm 20:** RCSP algorithm (Jin et al., 2019).

1. **Result**: RCSP feature vector 2. Import and preprocess the EEG signal 3. Apply Z-score normalization to the signals to normalize the mean of all the data to zero and the standard deviation to 1 4. Compute correlation coefficients between the channels. Use Pearson's correlation coefficient, as defined in Equation (40) (40) $$\begin{array}{l} 0<P\left(X,Y\right)=\;\frac{1}{n-1}\overset{n}{\underset{i=1}{\sum }}\left(\frac{X_{i}-\bar{X}}{\sigma_{X}}\right)\left(\frac{Y_{i}-\bar{Y}}{\sigma_{Y}}\right)<1, \end{array}$$ where *X* and *Y* are the observable variables, *n* is the number of observations, $\bar{X}$ and $\bar{Y}$ are the means of the observable variables, and σ_*Y*_ and σ_*X*_ are the standard deviations of the variables (Jin et al., 2019) 5. From the correlation coefficient matrices, extract the means from each row 6. Locate the row with the highest mean, and denote it as row *i*. This row is important as it is highly correlated with other channels 7. Select the channels that appear most often to move forward with 8. Apply Equations (36–39) to extract the RCSP features 9. Form the feature vector

This algorithm differs from the traditional CSP method by the introduction of two regularization parameters, α and β, which are used to create regularized average spatial covariance matrix (Jin et al., 2019):

(36) $$\begin{array}{l} C_{i}^{class}=\frac{E_{i}E_{i}^{T}}{trace\left(E_{i}E_{i}^{T}\right)}, \end{array}$$

where $C_{i}^{class}$ is the normalized covariance matrix (Jin et al., 2019).

(37) $$\begin{array}{l} {\overset{︷}{C}}_{i}^{class}=cov\left(E_{i}^{T}\right) \end{array}$$

where ${\overset{︷}{C}}_{i}^{class}$ is the pairwise covariance matrix (Jin et al., 2019), and *cov* is the function to calculate the pairwise covariance of each channel (Jin et al., 2019).

(38) $$\begin{array}{l} P^{class}\left(\alpha \right)=\frac{\left(1-\alpha \right)\sum_{i=1}^{N_{tr}}C_{i}^{class}+\alpha \sum_{i=1}^{N_{tr}}{Ĉ}_{i}^{class}}{N_{tr}} \end{array}$$

(39) $$\begin{array}{l} Q^{class}\left(\alpha ,\beta \right)=\left(1-\beta \right)P^{class}+\frac{\beta }{N_{s}}trace\left(P^{class}\right)I \end{array}$$

## 6. Discussions and conclusions

Through this review, we have studied and analyzed various techniques of EEG feature extraction from the time domain, frequency domain, decomposition domain, joint time-frequency domain and the spatial domain. Signal representation is best in the decomposition and joint time-frequency domains, when compared to the results from the time and frequency domains independently; however, various papers conclude that the spatial domain is ultimately the most powerful when it comes to EEG analysis and feature extraction (Dornhege et al., 2006; Jin et al., 2019; Khan et al., 2019; Reddy et al., 2019; Rashid, 2020).

In each review, the applications in ML, BCI-technology, assistive technology, disease diagnosis and more were also discussed. It has been thoroughly shown that the features extracted from EEG signals can be used and integrated into ML-pipelines successfully. The reader should note, however, that when developing a robust feature extraction pipeline, it must generate robust features and perform dimensionality reduction of the data prior to integration with an ML model (Krishnan and Athavale, 2018).

The review starts by analyzing one-dimensional feature extraction domains. Well-known methods in the time domain like the AR modeling, FD *via* Higuchi algorithm technique, statistical feature extraction, and detrended fluctuation analysis were reviewed. Time domain methods are commonly used as they are shown to have fast processing,

however they do not always yield the most relevant and robust features. Most time domain methods are also computationally inexpensive, like the extraction of statistical features from EEG signals. This means that they don't typically optimize the representation of the underlying EEG signals. This is also true for most frequency domain methods as well. This is because the time domain and frequency domain alone cannot capture sufficient detail and information in their features independently while ignoring the other domain. This comes back to the non-linear and non-stationary characteristics that EEG, and physiological signals as a whole, carry (Krishnan, 2021).

In the frequency domain, the FT, PSD, BP and HHT methods of feature extraction were studied and evaluated. High accuracies were shown to be achieved with these methods, like with band power analysis, however they can be computationally expensive (Mandel et al., 2009). Furthermore, these methods become less reliable when there are sudden shifts in frequency, which are characteristic of EEG signals (Azlan and Low, 2014).

In the decomposition domain, classical methods like the wavelet transform and empirical mode decomposition were studied, alongside other lesser-known methods like the AHD and LCSD. These methods are more adaptive (Taran and Bajaj, 2018), efficient and accurate (Liu et al., 2017; Ieracitano et al., 2020), but can be computationally slower (Liu et al., 2017; Ieracitano et al., 2020). The DWT was shown to have time-varying scale (inversely related to frequency) representation, overcoming the previously discussed limitation in the independent time and frequency domains. This is further overcome in the joint time-frequency domain, when multi-dimensional feature extraction begins.

The joint time-frequency domain looks at the features from both the time and frequency domains, hence extracting more detail and information from the EEG signals. This leads to higher efficiency like with the STFT method (Haddad, 1993) and better performance accuracy (Rutkowski et al., 2013), like in the MP method. However, with these methods, a balance must be found for time and frequency resolution, as there is an evident tradeoff between the two; as one increases, the other decreases (Haddad, 1993; Rutkowski et al., 2013; Ha, 2019).

**Figure 8:**
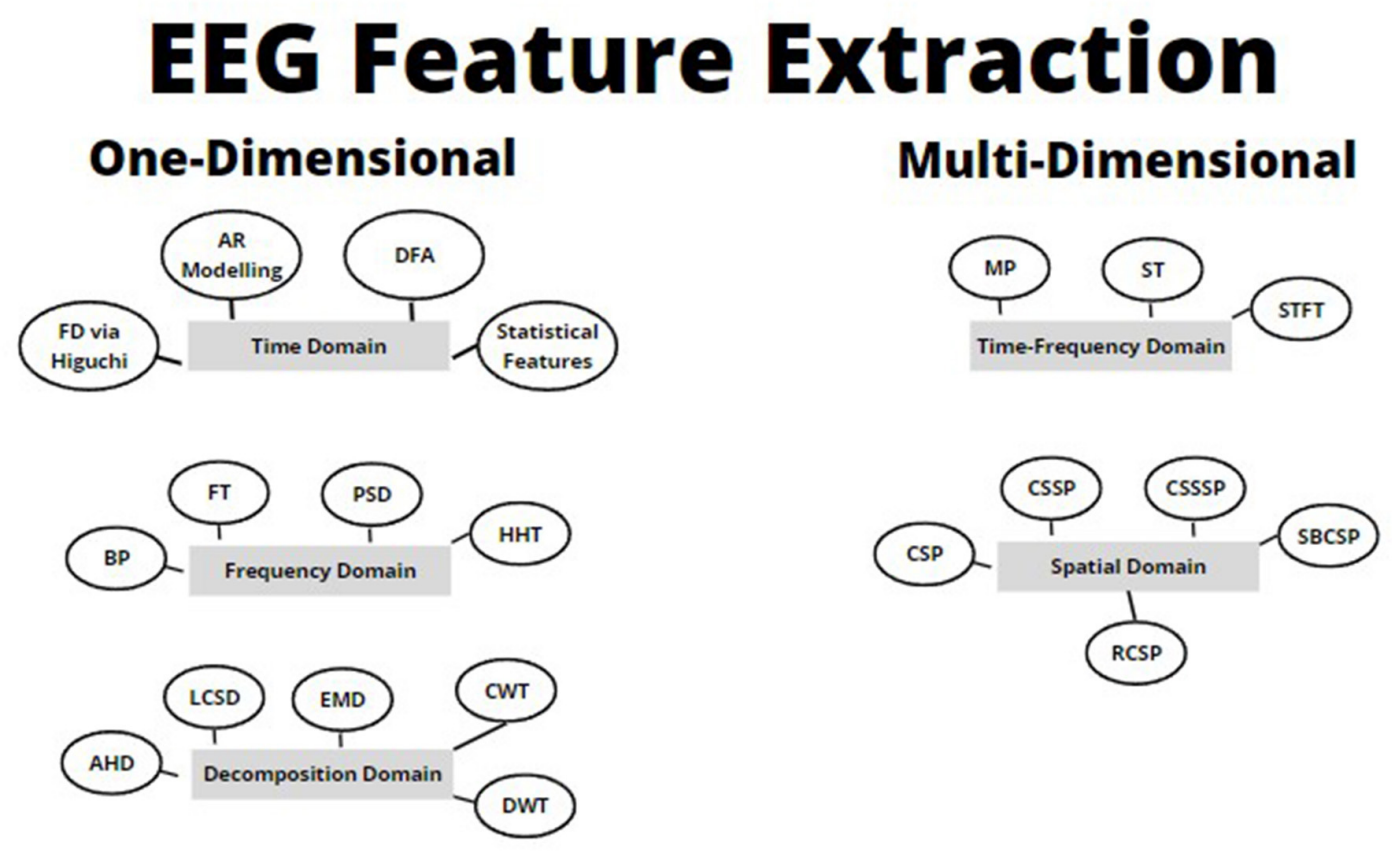
Summary of EEG feature extraction techniques.

The spatial domain, thought of as the most relevant domain for EEG feature extraction (Rashid, 2020), allows for the conversion of the brainwaves into a unique space for variance analysis (Reddy et al., 2019; Rashid, 2020). The broader method, CSP, has had many advancements proposed that were reviewed in this paper such as the CSSP, CSSSP, SBCSP, and the RCSP method. Each of these methods outperform the traditional CSP method, improve accuracy, and overcome CSP limitations; however, majority of these methods are computationally expensive (Dornhege et al., 2006; Jin et al., 2019; Khan et al., 2019; Reddy et al., 2019). All methods have been summarized in Figure 8 for quick reference purposes.

Each method summarized in Figure 8 has its own sets of pros and cons (refer to Tables 1–5), which will need to be weighed by the reader during implementation and testing. Based on the analysis of the various methods, it is clear that decomposition, time-frequency, and spatial domains provide the best representation on average of EEG signals, however it still is important to consider the methods in the other domains depending on the application or problem that you are trying to solve.

**Table 1:** Summary of time-domain feature extraction methods for EEG.

**Method**	**Sample applications**	**Advantages**	**Disadvantages**
AR modeling (Lawhern et al., 2012; Zhang et al., 2015; Chai, 2017)	EEG artifact classification Mental task classification Driver fatigue classification	Models peak spectra which are characteristic of EEG signals (high spectral resolution) All-pole model, meaning it is efficient for resolving sharp changes in the spectra	Challenging to choose model order; if too low, it will not represent the data well, and if it is too high, it will include noise
Fractal dimension (FD) *via* Higuchi algorithm (Kaur et al., 2018)	Emotion identification	Efficient method of feature extraction for non-stationary and non-linear data	The accuracy of this method has not been optimized
Statistical features (Picard et al., 2001)	Enhancement of BCI-machine intelligence systems with emotional intelligence	Simple implementation and computationally inexpensive	Statistical features can be extracted for many types of data, and may not always be the best choice for physiological signals
Detrended fluctuation analysis (Mumtaz et al., 2015)	Classification of patients with MDD	Efficiently discriminates MDD patients from healthy controls, allowing for diagnosis based on EEG data only	There is a small sample size constraint such that the results should not be generalized to a wider population

**Table 2:** Summary of frequency-domain feature extraction methods for EEG.

**Method**	**Sample applications**	**Advantages**	**Disadvantages**
Fourier transform (Delimayanti et al., 2020)	Sleep-stage/brainwave classification	Easy to implement, computationally inexpensive, and fast	Does not have excellent spectral estimation and cannot be used for the analysis of short EEG signals
Power spectral density (Chakladar and Chakraborty, 2018; Boonyakitanont et al., 2020; Delimayanti et al., 2020)	Cursor movement in BCI systems	Computationally inexpensive Versatile features available for increased accuracy	Difficulty examining non-stationary signals
Band power (Mandel et al., 2009)	BCI wheelchair control	High accuracy achieved	Computationally extensive as the feature extraction and classification need to be repeated for every 0.1 s of the EEG signal
Hilbert-Huang transform/Hilbert transform (Azlan and Low, 2014)	Schizophrenia disease detection	Has the ability to determine instantaneous frequency and power of a signal Ability to analyze nonlinear and nonstationary signals Retains the time information from time-frequency analysis	Tendency to generate uncertain results when there are sudden shifts in frequency in the time-series signal

**Table 3:** Summary of decomposition-domain feature extraction methods for EEG.

**Method**	**Sample applications**	**Advantages**	**Disadvantages**
Adaptive Hermite decomposition (Taran and Bajaj, 2018)	Drowsiness detection	Adaptive method, allowing for a more accurate detection	Challenge to find the optimal selection of order and dilation factor
Local characteristic-scale decomposition (Liu et al., 2017)	Motor imagery EEG signal classification	Efficient in redundant feature reduction	Time consumption is an on-going issue
Continuous wavelet transform (Ieracitano et al., 2020)	Automatic classification of EEG—dementia patients	High classification accuracy Provides information on how the frequency content changes over time	Computationally slower than the DWT
Discrete wavelet transform (Guo et al., 2015)	P300 event-related potential (ERP) Detection for BCIs	Optimal resolution in both the time and frequency domain Improved computational speed	Adaptive mother wavelet identification required for subject-to-subject analysis
Empirical mode decomposition (Zhang et al., 2008)	EEG denoising and preprocessing	Adaptive and highly efficient Results in an improved spectrum resolution Well suited for non-stationary and non-linear signals	Choosing the correct features to extract from the IMFs is challenging and requires more data-driven research

**Table 4:** Summary of time-frequency domain feature extraction methods for EEG.

**Method**	**Sample applications**	**Advantages**	**Disadvantages**
Short-time Fourier transform (Haddad, 1993; Rutkowski et al., 2013; Ha, 2019)	Motor imagery EEG signal classification for BCIs	Efficient detection of modal frequencies of linear time-invariant systems and their time localization	Cannot represent various resolutions due to the fixed window length Poor time-frequency resolution
S-transform (Rutkowski et al., 2013)	Classification of EEG signals—epilepsy	Uses a variable-length Gaussian window Acts as a phase-corrected wavelet transform	High computation complexity Lower degree of clarity that other distribution functions
Matching pursuits (Rutkowski et al., 2013)	Classification of EEG signals—Epilepsy and other neurological disorders	Method is flexible when compared to other leading approaches High discrimination performance	Greedy and computationally expensive method

**Table 5:** Summary of spatial-domain feature extraction methods for EEG.

**Method**	**Sample applications**	**Advantages**	**Disadvantages**
Common spatial pattern (Reddy et al., 2019; Rashid, 2020)	Oscillatory activity-based BCIs	Contributed to the improvement of Oscillatory Activity (OA)-BCI performance	Neglects the frequency information that is necessary for OA Due to the subject-specific optimal frequency band, it cannot achieve an ideal and efficient performance Performance suffers when non-discriminative rhythms with overlapping frequencies interfere
Common spatio-spectral pattern (Reddy et al., 2019)	EEG-based regression problems in BCIs	Overcomes the limitations the pure CSP algorithm faces	Computationally expensive
Common sparse spatio-spectral patterns (Dornhege et al., 2006)	Improvement of brain-computer interfacing	For the most part, the CSSSP algorithm outperforms its predecessor, the CSSP algorithm	With increasing *T*, the complexity (of the frequency filter) must remain under control to avoid overfitting
Sub-band common spatial patterns (Khan et al., 2019)	Multiclass EEG motor-imagery classification	Increased accuracy	Further research required for optimal channel selection
Regularized common spatial pattern (Jin et al., 2019)	Channel selection for motor imagery based BCI systems	Shown to improve classification accuracy through selection of relevant channels Minimized time complexity and maximized efficiency of feature extraction	Computationally expensive

## 7. Future work

There is much room for improvement in the methods discussed. Much of them only provided an accuracy in the range of 70%−80% which is not always sufficient depending on the application. Thus, the methods can be enhanced, and expanded upon for the use of real-time BCI applications, including assistive technology and disease diagnosis systems. As the health-technological revolution continues, we will be required to innovate in this regard. The real-time systems need to be improved so that the accuracy of the results rival that of the more robust yet computationally expensive methods such that they can be clinically accepted methods in the future. Furthermore, EEG signals are multi-channel signals due to the method of acquisition (refer to Figure 1). This leads to a higher degree of data. There are some methods discussed in this paper that went into optimized channel selection. If channel selection is further introduced into the methods discussed, it very well may increase the efficiency and accuracy of the systems, while reducing computation time and complexity. This would greatly benefit the domain of BCI systems, assistive technology and neurological disease diagnosis.

## Author contributions

AS: writing—original draft preparation, methodology, software, data curation, and investigation. SK: conceptualization, supervision, validation, and writing—reviewing and editing. Both authors contributed to the article and approved the submitted version.
